# Indents

**Published:** 1904-09-15

**Authors:** 


### INDENTS.

☞ **Brief Articles of Technical or Scientific Value will receive notice and comment. Contributions invited.**

**Investment.** Plaster of paris, two parts, and Portland cement, one part, makes the quickest setting and most reliable investment I have ever used.—Dr. S. Moyer, *Review.*

**Chloro-percha in Crowns.** Before setting crowns or bridges, flow a thin layer of chloro-percha inside your crown or abutment caps before setting with cement. It facilitates removal in case of necessity.—*Summary.*

**Gauze Drains Contra-indicated in Pus Cavities.** Never forget the fact that gauze will drain serum or very fluid discharges, but not pus. Hence the filling up of an abscess cavity with gauze is the surest possible way of blocking-in the secretion and favoring sepsis.—*International Journal of Surgery.*

**Pyorrhea.** In treatment of pyorrhea alveolaris, one of our university professors advises: "First the old sulphuric acid treatment (clearing out all germ life in the pockets), following with Phillips' milk of magnesia sprayed into the pockets. This latter continued as daily treatment."—*Exchange.*

**Low-fusing Enamels.** Mix a small amount of Brewster enamel body with the XX body and get a modified color which will fuse at a lower temperature, or rather in less time than the body alone. It may be advantageously used in building up specially-difficult inlays in sections as it were.—Dr. Thompson, in *American Journal.*

**Brooch in a Tooth affects Eyesight.** It is reported from Port Huron, Mich., that a young lady has been gradually losing her sight, and has recovered it after having a tooth removed which contained a nerve broach left in the canal and sealed in with a filling. It seems incredible, yet it may be true.—Item in *Digest.*

**Nail-cleaning Liquid.**

| | |
|---|---|
| Tartaric acid | 1 dr. |
| Myrrh tincture | 1 dr. |
| Cologne water | 2 drs. |
| Water | 3 ozs. |

Dissolve the acid in water; mix the myrrh tincture and cologne, and add to the acid solution. Dip the nails in this solution, wipe, and polish with chamois skin.

**Skin Varnish.** Used by surgeons to coat the fingers so as to minimize the transferance of disease-germs. The varnish is removed after completing the examination of the patient.

| | |
|---|---|
| Copal | 2 parts |
| Venice turpentine | 4 parts |
| Ether | 100 parts |
| Collodion | 100 parts |
| Acetone | 8 parts |

—*Pacific Drug Review.*

**Prevention of Adhesion of Impression Material.** To prevent plaster or other material adhering to the teeth, the patient may be requested to take a mouthful of Phillips' milk of magnesia, and hold it for a few minutes previous to insertion of material to be employed. This will cleanse the teeth and mucous surfaces of adherent secretions, leaving a slight film of magnesia, preventing sticking of material in hardening. A clear, sharp impression results.—*Exchange.*

**An Aid in Removal of Gum.** In cases of the gum covering a cavity in which there may be, possibly, an exposed pulp to treat, much pain and annoyance will be saved by first applying trichloracetic acid, which destroys all organic matter, but not inorganic. It does not matter if it should touch the pulp. In a quarter of an hour you will be able to cut away without any pain. It is very useful in case of difficulty erupting third molars; just put a few crystals between the gum and the tooth.--*Cosmos*.

**Quick Separation Contra-indicated in Teeth to be Filled with Amalgam.** When amalgam is used quick wedging is contra-indicated in any but small fillings. Unless the teeth are kept apart sufficiently long to overcome the tendency to immediately resume their normal position, any contouring will be destroyed; possibly, indeed, the filling may be broken up and disintegrated by the teeth springing together as soon as the wedge is removed.—D. Linley Palmer, *Brief*.

**Restoring Tarnished Gold.**

| | |
|---|---|
| Sodium bicarbonate | 20 ozs. |
| Chlorinated lime | 1 oz. |
| Common salt | 1 oz. |
| Water | 16 ozs. |

Mix well and apply with a soft brush. A very small quantity of the solution is sufficient for effecting the desired purpose, and it may be used either cold or lukewarm. Plain articles may be brightened equal to new by putting a spot or two of the liquid upon them from the stopper of the bottle and lightly brushing over the surface with fine tissue paper until sufficiently dried off to accomplish the object intended.

**Modern Dental Outfit Dangerous.** Dr. J. F. Wagner, of Seattle, Wash., writing in the *Gazette*, states that on several occasions, his patients received a severe electric shock while he was inserting metal fillings. On investigation he found that when his foot touched the rheostat or controller of his electric engine, with the patient's feet resting on the metal foot-piece of the chair, the fountain cuspidor running, the patient received an electric shock. An electrician of his acquaintance warned him that if conditions should be just right the patient might be seriously injured.—*Digest.*

**Saliva as a Factor in the Cure of Disease.** Bergmann (*Therapie der Gegenwart*) styles the gargle therapeutic trifling and inefficient for any real purpose. He thinks, on the other hand, that saliva reaches every part of the throat and can be made a vehicle for therapeutic application. To increase the flow of saliva he has chewing tablets and advocates their use in dyspepsia, obesity and edema. He has a special medicated and non-medicated tablet for each form. The alkaline saliva is especially beneficial in acid dyspepsia. In obesity and edema the accumulating saliva is expelled in large amounts and the results are surprisingly favorable.—*Digest,*

**Guide For Fusing Porcelain.** Dr. A. O. M. Le Cron, of St. Louis, has tested the var'ous porcelains and devised a metallic alloy which will melt at the exact fusing point of the porcelain. This will enable one to know exactly when his baking is done without watching the furnace constantly. Dr. Roach, of Chicago, has invented a timing arrangement which will cut off the current at the proper fusing time. This is accomplished by placing in a special slot in the furnace a lead bullet, which is fused at the temperature which melts lead, and this molten lead runs down into a pan on a lever which starts a clock, which can be set to run a certain number of seconds or minutes, when it cuts off the current automatically.

**Nature of Shock.** Frank P. Vale, in August *New York Medical Record*, after careful study, concludes that shock is not due to the exhaustion of any one nerve center, or paralysis of any one system; but every tissue and organ in the body is affected through the nervous system. There is an extravasation of lymph into the tissues, and a lowering of the vasomoter tone. Enfeebled heart action is a secondary to lowered blood pressure. The wan and pinched expression is due to empty peripheral vessels and feeble circulation or loss of blood from injury. Lessened secretions are due to lowered blood pressure. Loss in temperature is due to general arrest of circulation and metabolic processes. Shallow respiration is in accord with the phenomena of aerated venous blood. Inspired consciousness is due to the profound impression on the nerve centers and the feeble circulation.

**Suicide by Carbolic Acid.** Health Commissioner Darlington, of New York City, gives out some startling figures relating to the use of carbolic acid by suicides in the big city, and calls upon druggists to be more careful in dispensing the poison. In 1903, 805 men and women in the five boroughs of this city killed themselves, and of these, 337 used carbolic acid, a trifle more than 40 percent of the total number. The records show a steady increase in the number and proportion of suicides by carbolic acid.

It has been suggested that an ordinance be enacted, similar to the one now in force in Chicago, providing generally that carbolic acid may be sold only in five percent solution, unless called for in a prescription.

The question presents itself whether any such enactment would result in decreasing the number of suicides, presuming that to be the purpose. Anybody so desperate as to attempt to use carbolic acid to destroy himself, after all that has been printed of the horrible agonies of such a death, would be likely, if thwarted in his original attempt, to find some other way of accomplishing his end. When the carbolic acid ordinance shall have been in effect for a year in Chicago, other municipalities will know whether or not similar ordinances are likely to be worth while.—*Merck's Report.*

